# Transanal Minimally Invasive Surgery (TAMIS) in Qatar: initial experience

**DOI:** 10.1186/s12893-020-00797-6

**Published:** 2020-06-19

**Authors:** A. Abutaka, A. Ahmed, M. Abunada, M. Kurer

**Affiliations:** Colorectal Surgery Section, Department of Surgery, Hamad General Hospital, P.O Box 3050, Doha, Qatar

**Keywords:** TAMIS, Rectal tumors

## Abstract

**Background:**

Transanal Minimally Invasive Surgery (TAMIS) has revolutionized local excision of mid and high rectal lesions; benign or malignant. It is a technique that is developed as a hybrid between Transanal Endoscopic Microsurgery (TEM) and laparoscopic surgery for resection of rectal lesions.

**Methods:**

We retrospectively reviewed prospectively collected data on patients who underwent TAMIS for benign and early malignant rectal lesions between Jan 2015 and Sept 2019, at Hamad General Hospital, Doha, Qatar.

We assessed the following outcomes: feasibility, fragmentation of specimen, operative time, length of stay (LOS) post-operative complications, and margin negativity.

**Results:**

Seventeen consecutive patients underwent TAMIS for benign and malignant rectal lesions. The average length of stay (LOS) is 1.5 days (1–6 days). Seven patients had different types of benign adenomas, five patients had proven adenocarcinoma, three patients had well-differentiated neuroendocrine tumors, one patient with hyperplastic polyp, and one patient had inflammatory polyp. No fragmentation occurred or detected by histopathologic examination, except in a patient who had inflammatory polyp, where the lesion removed in two fragments.

**Conclusion:**

TAMIS procedure is feasible and safe even in a relatively low-volume colorectal unit. Using this tool, many patients can avoid unnecessary radical surgery. Therefore, we believe that TAMIS should form part of every specialized colorectal service repertoire. To our knowledge, this is the largest series in the gulf region.

## Background

Management of rectal lesions, benign or malignant, evolved with the improvement of surgical tools and techniques. Total mesorectal excision (TME) and proctectomy remain the gold standard curative procedure [1, 2]. Based on evidence from multiple large randomized controlled trials (RCTs), minimally invasive TME operations using laparoscopy and robotic systems have been implemented and used widely across the globe.

Rectal lesions are increasingly detected with increased screening and awareness. This is connected to improved tools of diagnosis and management of these lesions.

Local excision (LE) of benign and early malignant rectal lesions was traditionally described to remove lesions within 8 cm from the anal verge [3]. Higher lesions in the rectum rendered amenable for local excision using Transanal Endoscopic Microsurgery system described by Buess et al. more than three decades ago [4]. It became more attractive for many reasons; such as better functional outcomes, less morbidity, faster recovery and avoidance of radical resections [3].

Transanal Minimally Invasive Surgery (TAMIS) has revolutionized local excision of mid and high rectal lesions; benign or malignant. Since its introduction in 2009 by Atallah et al., this hybrid tool between single port laparoscopy and Transanal Endoscopic Microsurgery (TEM), has gained wider acceptance and popularity than TEM or the traditional Parks Transanal Excision (TAE), due to its superior surgical outcomes, shallow learning curve, and low cost, in addition to easier setup and more flexibility during the procedure [5].

## Methods

We retrospectively reviewed prospectively collected data on patients who underwent TAMIS for benign and early malignant rectal lesions between Jan 2015 and Sept 2019, at Hamad General Hospital, Doha, Qatar. All cases were discussed in colorectal multidisciplinary team (MDT) and the procedures were done by a single colorectal surgeon or under his direct guidance.

For malignant lesions, preoperative staging accomplished by CT scan and Rectal MRI. All cases were discussed in the tumor board before the procedure.

We assessed the following outcomes: feasibility, fragmentation of specimen, operative time, length of stay (LOS) post-operative complications, and margin negativity.

All patients underwent TAMIS in Lioyd-Davies position under general anesthesia (GA), except one patient who refused GA and had spinal anesthesia. We used GelPOINT path transanal access platform, and maintained pneumorectum to pressure of 15 mmHg with AirSeal system insufflator. As a standard operative preparation; all patients were positioned in Lloyd Davis position with a Bean Bag underneath the patient, in case laparoscopic access to the peritoneal cavity was to be required. All patients were given rectal phosphate enema approximately 4 h before the procedure. All patients received standard DVT prophylaxis. Prophylactic antibiotics were also administered at induction of anesthesia.

**Fig. 1:**
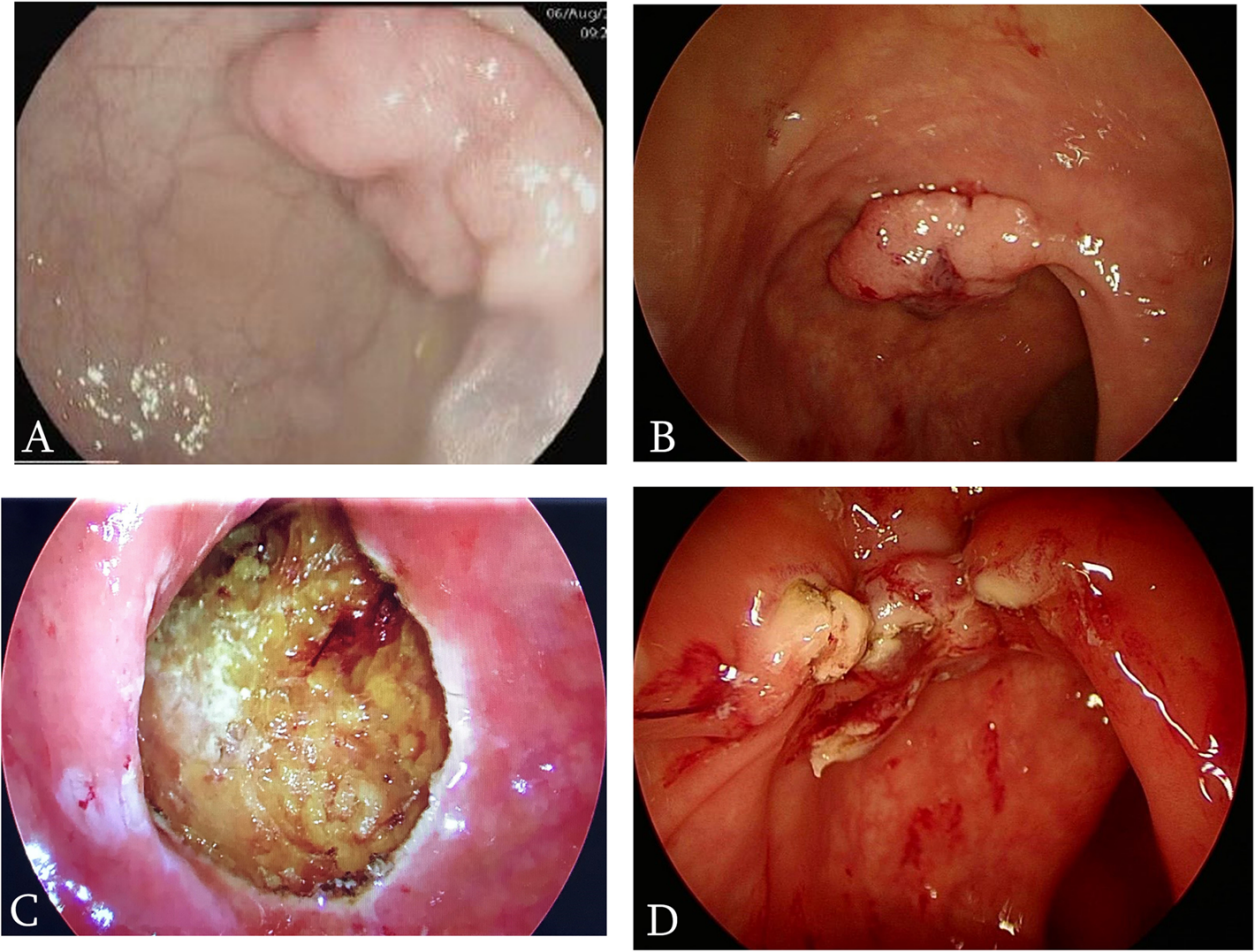
**a** Colonoscopic appearance of rectal sessile polyp. **b** TAMIS view of the same polyp. **c** the polyp post TAMIS resection before closure. **d** resection site post closure

We used the GelPOINT path transanal access platform (Applied Medical, Rancho Santa Margarita, CA), AirSeal port (Conmed, NY, USA) to establish and sustain pneumorectum, and 30 degrees scope. We always approximate the defect after excision with V-Loc™ Vicryl (Medtronic, Minneapolis, MN, USA) The specimen then sent oriented to the histopathology department (Fig. 1).

## Results

Seventeen consecutive patients underwent TAMIS for benign and malignant rectal lesions, in the period between Jan 2015 and September 2019. The patients’ population consisted of 6 women (34%) and 11 men (64%), with an average age of 52 years (28–88). Nine patients had an ASA score of 2, six patients of ASA score 3, and three patients with an ASA score of 1 (Table 1).

**Table 1:** Patients demographics

Patients, n	17
• Men	11 (64%)
• Women	6 (34%)
Age (years)	52 (28–88)
ASA Score	
• I	3
• II	9
• III	5

The average length of stay (LOS) is 1.5 days (1–6 days). Eleven out of the eighteen patients discharged on day 1 post operatively. One patient stayed 6 days because of post-operative hypotension which was managed conservatively.

The average operative time was 74.17 min (20–180 min). The average distance of the lesion from the anal verge is 7.47 cm (3–18 cm). Eight patients (47%) had the lesion > 7 cm from the anal verge.

Seven patients had different types of benign adenomas, five patients had proven adenocarcinoma, three patients had well-differentiated neuroendocrine tumors, one patient with hyperplastic polyp, and one patient had inflammatory polyp.

No fragmentation occurred or detected by histopathologic examination, except in a patient who had inflammatory polyp, where the lesion removed in two fragments. The average size of the excised lesions is 2.62 cm (1.2–7 cm). All resection margins were free, the nearest margin was 4 mm in a tubular adenoma specimen. Six patients had a discrepancy between the preoperative and the post-operative histopathology; one of the patients down-staged from polyp adenocarcinoma to polyp high-grade dysplasia. Another patient was diagnosed as moderately differentiated adenocarcinoma pre-operatively; which is located 5 cm from the anal verge, down-staged to benign lesion on final histopathology following TAMIS excision. Both of these patients were spared radical resections (Table 2).

**Table 2:** Tumor characteristics and histopathology for locally excised lesions

Distance from anal verge, cm (range)	7.41 (3–18)
Tumor size, cm (range)	**2.62** (1.2–7)
Benign	
• Hyperplastic	1
• Adenoma	6
• Inflammatory	1
Cancer	
• T0	6
• T1	1
• T2	4
Neuroendocrine Tumor	3
Discrepancy (pre-op to post-op)	
• Adenoma to Hyperplastic polyp	1
• Hyperplastic polyp to adenoma	1
• Adenoma to Polyp cancer	1
• Polyp cancer to polyp high-grade dysplasia	1
• Low-grade dysplasia to adenoma	1
• Polyp high grade dysplasia to polyp cancer	1
• Fragmentation	1
• Positive margins	None
• Procedure-related complications (30 days)	Bleeding not requiring transfusion (one patient) Intraoperative peritoneal perforation, repaired immediately (one patient)

In one patient, the peritoneum is entered during the excision of a tubular adenoma 10 cm from the anal verge. Laparoscopic closure of the peritoneum was achieved as well as transanal closure. The patient was discharged 2 days post operatively, with no complications.

There was one procedure-related complication during excision of a serrated adenoma located 10 cm from the anal verge, were the peritoneum was breached. Immediate laparoscopic repair was done, and the patient discharged home on day 2 post-operatively with no complications.

Another patient had minimal post-operative fresh bleeding per rectum which required no transfusion.

There were no other procedure-related immediate or 30-day complications in any of the other patients.

## Discussion

Transanal Minimally Invasive Surgery (TAMIS) is a technique that is developed as a hybrid between Transanal Endoscopic Microsurgery (TEM) and laparoscopic surgery for resection of rectal lesions. Adoption of the technique has spread widely due to availability of the laparoscopic tools and insufflators and the single-site port, as well as the shallow learning curve, and most importantly the comparable safety and oncologic outcomes.

NCCN guidelines have defined the lesions which are appropriate for local excision using any system: mobile rectal tumors, less than 3 cm in size, occupying less than one-third of the circumference of the bowel, not extending beyond the submucosa, with well to moderate differentiation, and low-risk histopathological features. Transanal local excision is not appropriate for rectal tumors with high-risk characteristics, including lymphovascular invasion, perineural invasion, and mucinous components [1].

We have demonstrated a congruent result of safety and oncologic outcomes using TAMIS compared to the existing literature and case series [6–8]. All our malignant lesion resections as well as NET resections were margin negative. The reported positive margin in a recent large series of 200 patients was 7% [9], and up to 2 out of 3 NET resections in some series [10] (Tables 3 and 4).

**Table 3:** Characteristics of individual cases

No	Age group / Sex	Location (cm)	Pre-operative HP	Indication	Tumor size (cm)	Post-operative HP	Need for further management
1	35–45/ S2	7	Tubulovillous adenoma with low grade dysplasia	Treatment	2.8 × 2.5	Tubulovillous adenoma	None
2	45–55/ S2	18	Serrated adenoma with low grade dysplasia	Treatment	1.5 × 0.5	hyperplastic polyp	None
3	55–65/ S1	5	Moderately differentiated adenocarcinoma	Treatment	2.2 × 1.7	pT0Nx adenocarcinoma	None
4	55–65/ S2	10	Serrated adenoma	Treatment	2.5 × 2.2	Serrated adenoma	None
5	55–65/ S2	10	Hyperplastic polyp	Treatment	2.5 × 2.2	Tubular adenoma	None
6	25–35/ S1	4	Well differentiated NET	Treatment	2.7 × 2.2 cm (tumor 4 mm)	Well differentiated NET	None
7	55–65/ S1	10	Tubulovillous adenoma	Treatment	2.3 (cancer 2 mm)	Polyp Moderately differentiated adenocarcinoma (Haggit’s 1)	None
8	85–95/ S1	3	Moderately differentiated adenocarcinoma (cT2 / early T3 N0 M0)	Treatment	3.9 × 2.3	Moderately differentiated adenocarcinoma (pT2)	None
9	55–65/ S2	8	Well differentiated NET	Treatment	1.2 × 0.6 mm	Well differentiated NET	None
10	35–45/ S1	4	Moderately differentiated invasive adenocarcinoma, arising in a tubular adenoma with high-grade dysplasia	Treatment	1.5 × 0.7	Tubular adenoma with high-grade dysplasia	None
11	65–75/ S2	3	Villous adenoma with at least high-grade dysplasia and suspicions cancer	Treatment	3 × 2 × 0.5	Moderately differentiated adenocarcinoma cT1N0M0	None
12	35–45/ S1	5	Well differentiated NET	Treatment	2.3 × 1.7 × 0.7 (nodule 0.8 cm)	Well differentiated NET	None
13	25–35/ S1	9	Low grade dysplasia	Treatment	7 × 5 × 4 cm	Villous adenoma	None
14	45–55/ S1	5	Tubulovillous adenoma with focal high-grade dysplasia	Treatment	3 × 2.5 × 1.5	Tubulovillous adenoma	None
15	35–45/ S1	10	Well differentiated adenocarcinoma on tubulovillous adenoma (incomplete colonoscopic removal)	Treatment	4.5 × 3.6 × 2	Residual well differentiated adenocarcinoma on tubulovillous adenoma (SM3)	None
16	45–55/ S1	5	None	Diagnostic and treatment	2.7 × 1.8 × 0.8 cm and 2.8 × 2 × 0.4 cm	benign inflammatory cloacogenic polyp/ mucosal prolapse.	None
17	55–65/ S1	10	Tubulovillous adenoma with focal high-grade dysplasia	Treatment	2.5 × 1.7 × 1.8 cm	Moderately differentiated invasive adenocarcinoma with mucinous component in a background of tubulovillous adenoma with focal high grade dysplasia. Kikuchi SM2	None

**Table 4:** Characteristics of malignant lesions excised using TAMIS

No	Age group/sex	Location (cm)	Pre-operative HP	Colonoscopy findings	MRI findings	Indication	Tumor size (cm)	Post-operative HP	Need for further management
1	55–65 / S1	5	Moderately differentiated adenocarcinoma (reaching inked margin)	Sessile polyp in the rectum (snared)	No gross lesion found	Treatment	2.2 × 1.7	pT0, no residual; tumor	None
2	55–65 / S1	10	Tubulovillous adenoma	Flat polyp	Lesion in mid-rectum confined to muscularis propria	Treatment	2.3 (cancer 2 mm)	Polyp Moderately differentiated adenocarcinoma (Haggit’s 1)	None
3	85–95 / S1	3	Moderately differentiated adenocarcinoma (cT2 / early T3 N0 M0)	Ulceroproliferative lesion in the rectum	No MRI. EUS showed T2 / early T3	Treatment, patient unfit for radical resection	3.9 × 2.3	Moderately differentiated adenocarcinoma (pT2)	None
4	35–45 / S1	4	Moderately differentiated invasive adenocarcinoma, arising in a tubular adenoma with high-grade dysplasia	2 cm rectal lesion (infiltrating the mucosa)	Definite polypoidal lesion at the lower rectum T1/T2 with thickened CRM	Treatment	1.5 × 0.7	Tubular adenoma with high-grade dysplasia	None
5	75–85 / S2	3	Villous adenoma with at least high-grade dysplasia and suspicions cancer	Rectal mass with query malignant features	No MRI. EUS: T3 lesion	Treatment	3 × 2 × 0.5	Moderately differentiated adenocarcinoma cT1N0M0	None
6	35–45 / S1	10	Well differentiated adenocarcinoma on tubulovillous adenoma (incomplete colonoscopic removal)	Large rectal polyp 10 cm from anal verge, with broad base. Removed incompletely in fragments	Lesion in the upper rectum could not be clearly appreciated because of metallic clips placed after polypectomy	Treatment	4.5 × 3.6 × 2	Residual well differentiated adenocarcinoma on tubulovillous adenoma (SM3)	None
7	55–65 / S1	10	Tubulovillous adenoma with focal high-grade dysplasia	Sessile polypoid lesion with central depression, about 1.5 cm in size, at 10 cm from anal verge	Right posterolateral polypoidal wall thickening measuring approximately 15 mm, with central hyperintensity; no significant diffusion restriction or hyperenhancement. No extension beyond the muscularis.	Treatment	2.5 × 1.7 × 1.8 cm	Moderately differentiated invasive adenocarcinoma with mucinous component in a background of tubulovillous adenoma with focal high grade dysplasia. Kikuchi SM2	None

Our series showed that despite the fact that a relatively small number of patients dispersed over a period of nearly 5 years, the surgical outcomes, operative time, and quality of specimens did not compare unfavorably. One of our patients had a lesion 10 cm from anal verge with malignant features of T3 tumor by MRI, which was difficult to diagnose using colonoscopy due to superficial biopsy. TAMIS used in this patient to obtain adequate biopsies, which confirmed adenocarcinoma enabling the patient to receive neoadjuvant chemoradiotherapy.

## Conclusions

TAMIS procedure is feasible and safe even in a relatively low-volume colorectal unit. Using this tool, many patients can avoid unnecessary radical surgery. Therefore, we believe that TAMIS should form part of every specialized colorectal service repertoire. To our knowledge, this is the largest series in the gulf region.

## Data Availability

All data generated or analysed during this study are included in this published article.

## Abbreviations

TAMIS: Transanal Minimally Invasive Surgery
TEM: Transanal endoscopic microsurgery
TAE: Transanal excision
LE: Local excision
MDT: Multidisciplinary team
NET: Neuroendocrine tumor
LOS: Length of stay
ASA: American College of Anesthesiologists Physical Status Classification
RCT: Randomized controlled trial
CT scan: Computerized tomography scan
MRI: Magnetic resonance imaging
